# The Genetic Basis of Baculum Size and Shape Variation in Mice

**DOI:** 10.1534/g3.116.027888

**Published:** 2016-03-01

**Authors:** Nicholas G. Schultz, Jesse Ingels, Andrew Hillhouse, Keegan Wardwell, Peter L. Chang, James M. Cheverud, Cathleen Lutz, Lu Lu, Robert W. Williams, Matthew D. Dean

**Affiliations:** * Molecular and Computational Biology, Department of Biological Sciences, University of Southern California, Los Angeles, California 90089; † University of Tennessee, Health Science Center, Memphis, Tennessee 38163; ‡ Texas A & M, Veterinary Medicine and Biomedical Sciences, College Station, Texas 77845; § The Jackson Laboratory, Bar Harbor, Maine 04609; ** Loyola University, Department of Biology, Chicago, Illinois 60626

**Keywords:** baculum, sexual selection, shape, size

## Abstract

The rapid divergence of male genitalia is a preeminent evolutionary pattern. This rapid divergence is especially striking in the baculum, a bone that occurs in the penis of many mammalian species. Closely related species often display diverse baculum morphology where no other morphological differences can be discerned. While this fundamental pattern of evolution has been appreciated at the level of gross morphology, nearly nothing is known about the genetic basis of size and shape divergence. Quantifying the genetic basis of baculum size and shape variation has been difficult because these structures generally lack obvious landmarks, so comparing them in three dimensions is not straightforward. Here, we develop a novel morphometric approach to quantify size and shape variation from three-dimensional micro-CT scans taken from 369 bacula, representing 75 distinct strains of the BXD family of mice. We identify two quantitative trait loci (QTL) that explain ∼50% of the variance in baculum size, and a third QTL that explains more than 20% of the variance in shape. Together, our study demonstrates that baculum morphology may diverge relatively easily, with mutations at a few loci of large effect that independently modulate size and shape. Based on a combination of bioinformatic investigations and new data on RNA expression, we prioritized these QTL to 16 candidate genes, which have hypothesized roles in bone morphogenesis and may enable future genetic manipulation of baculum morphology.

The rapid divergence of male genitalia is an almost universal evolutionary pattern among sexually reproducing organisms (Hosken and Stockley 2004; Eberhard 2009, 1985). Rapid evolution extends to the baculum, a bone in the penis of many mammalian species. Even a cursory examination of over 200 illustrations by Burt (1960) reveals a bewildering array of baculum diversity in size, shape, amorphousness, symmetry, and even the number of bones and patterns of branching. Mammalogists have long recognized the utility of the baculum in identifying species that are otherwise morphologically indistinguishable (*i.e.*, Herdina *et al.* 2014; Thomas 1915), even positing that baculum morphology reinforces reproductive isolation between species (Patterson and Thaeler 1982, but see Good *et al.* 2003).

The selective forces that drive the evolutionary novelty of the baculum are likely to include a combination of female choice, male-male competition, and conflict between male and female reproductive interests (House and Simmons 2003, 2005; Eberhard 2009, 1996, 2000; Breseño and Eberhard 2009; Arnqvist 1998; Arnqvist and Rowe 2005; Perry and Rowe 2012; Rönn *et al.* 2007; Rowe and Arnqvist 2012; Schulte-Hostedde *et al.* 2011; Hosken and Stockley 2004; Tasikas *et al.* 2009; Patterson 1983; Patterson and Thaeler 1982; Long and Frank 1968; Ramm 2007; Brennan *et al.* 2007, 2010; Arnqvist and Danielsson 1999; Higginson *et al.* 2012). The enormous interspecific divergence in baculum morphology, coupled with relatively low levels of intraspecific variation (*e.g.*, Ramm *et al.* 2010; Klaczko *et al.* 2015), suggests that male genitalia are the targets of recurrent adaptive evolution.

Several studies have linked baculum characteristics with male reproductive success. In seminatural enclosures of multiple male and female house mice, males with a wider baculum sired both more and larger litters (Stockley *et al.* 2013). Similarly, in wild populations and experimental populations subjected to higher levels of sperm competition, males developed bacula that were significantly wider than males from low sperm competition populations (Simmons and Firman 2014). Dominant males tend to develop wider bacula (Lemaître *et al.* 2012), suggesting additional links between baculum morphology and presumably adaptive behavioral phenotypes. Since sperm competition is a regular feature of mouse mating ecology (Dean *et al.* 2006; Firman and Simmons 2008), baculum size and shape are probably important components of fitness in natural populations. In mice, the baculum resides in the distal portion of the glans penis which enters the vagina during copulation (Rodriguez *et al.* 2011). Intromission may be an important aspect of “copulatory courtship,” which influences fertilization success beyond delivery of ejaculate (Adler and Toner 1986; Matthews and Adler 1978; Toner and Adler 1986; Toner *et al.* 1987; Diamond 1970), and it is possible that the baculum plays a role in these processes.

The puzzling diversity of baculum morphology and its link to male reproductive fitness demand more attention. A genetic dissection of the ultimate sources of variation in baculum morphology has been hindered by two main obstacles. First, bacula lack true landmarks such as sutures, foramina, or processes. As a result, most studies summarize baculum complexity with simple length, width, and/or weight measurements (Long and Frank 1968). Some studies have captured baculum outlines using more modern computational techniques (Simmons and Firman 2014), but ideally, baculum size and shape would be captured in three dimensions. Here, we overcome this challenge using tools in computational geometry and geometric morphometrics to measure bacula without reliance on true landmarks, which should be applicable to many different biological structures.

Second, although variation in baculum morphology is heritable (Simmons and Firman 2014), no study to date has mapped the genetic basis of variation in size or shape. Both genetic causes and molecular mechanisms underlying its rapid evolution are uncharted. The rapid evolutionary divergence discussed above predicts a relatively simple genetic architecture in which mutations in a few key loci lead to large changes in morphology. Furthermore, we might predict that size and shape would be modulated by different loci, so that divergence in one aspect is uncoupled from divergence in the other. Here, we unite our newly developed morphometric methods with the power of mouse genetics in a quantitative genetics framework, using two genetic models: a family of recombinant inbred lines (RILs) known as the BXDs (Taylor *et al.* 1973, 1999; Peirce *et al.* 2004), and a family of advanced intercross lines (AILs) known as the LGxSM (reviewed in Nikolskiy *et al.* 2015).

Our study makes several advances toward understanding the genetic basis of baculum size and shape. We demonstrate that variation in baculum size and shape is heritable, and is controlled by a small number of QTL with comparatively large effects. We identified different QTL affecting size *vs.* shape that, when combined with new data on gene expression and bioinformatic filtering, highlight several compelling candidate genes. Future molecular analyses should eventually lead to a better understanding of genetic mechanisms of recurrent adaptive morphological evolution.

## Materials and Methods

### Specimens

All protocols and personnel were approved by USC’s Institute for Animal Care and Use Committee (protocol #11394). Our study was based on two powerful mouse genetic models, the BXD RILs and the LGxSM AILs. Most mice were already euthanized and frozen as part of unrelated research programs; a few were raised in-house or ordered through common vendors, then euthanized via carbon dioxide exposure followed by cervical dislocation.

The BXD RILs began in the 1970s with approximately 30 RILs (Taylor *et al.* 1973, 1999) and were expanded to ∼150 lines (Taylor *et al.* 1999; Peirce *et al.* 2004; R. W. Williams, unpublished results). All strains have been genotyped at over 3000 informative markers (www.genenetwork.org). Each BXD RIL began as a cross between two classical inbred strains, C57BL/6J (B6) and DBA/2J (D2). BXD1 through BXD42 were then maintained via brother-sister mating. BXD43 and higher were maintained through nonsib matings until generation 8–12 to accumulate larger numbers of recombinations and then inbred, making them an advanced intercross-rederived RIL family (Peirce *et al.* 2004), to create homozygous strains that on average have 50% B6 and 50% D2 genome, but with a different 50% segregating across different BXD RILs dependent upon the randomness of recombination during inbreeding. Individuals from the same BXD RIL are essentially genetically identical, and can be considered biological replicates of the same genotype. We sampled males that were 60–200 d old since the baculum is fully developed at this age (Glucksmann *et al.* 1976). The genomes of B6 and D2 have been nearly completely sequenced (Keane *et al.* 2011; Waterston *et al.* 2002; Wang *et al.* 2016), providing power to followup investigations of QTL.

The LGxSM AILs began from crosses between the LG/J and SM/J strains, originally selected over many generations for large and small body size, respectively (Goodale 1938; MacArthur 1944). LGxSM AILs were not intentionally inbred, but rather maintained through random breeding of unrelated individuals. Approximately 100 families are created each generation by pairing males and females that are not full siblings. Each generation accumulates more recombination events that break up the genomes of the two parental strains while avoiding inbreeding. Published studies of the LGxSM AILs exist at the F2, F8, and F34 generations (Norgard *et al.* 2011; Parker *et al.* 2011, 2014, 2012; Cheng *et al.* 2010). As part of unrelated research, several hundred individuals of the F43 and F44 generations were genotyped at thousands of markers (J. M. Cheverud, unpublished results). These F43 and F44 mice were sampled here. Unfortunately, many of the frozen carcasses were missing the penis as a result of prior necropsy, and only 144 males could be sampled. The parental LG/J and SM/J genomes have been sequenced (Nikolskiy *et al.* 2015).

**Figure 1:**
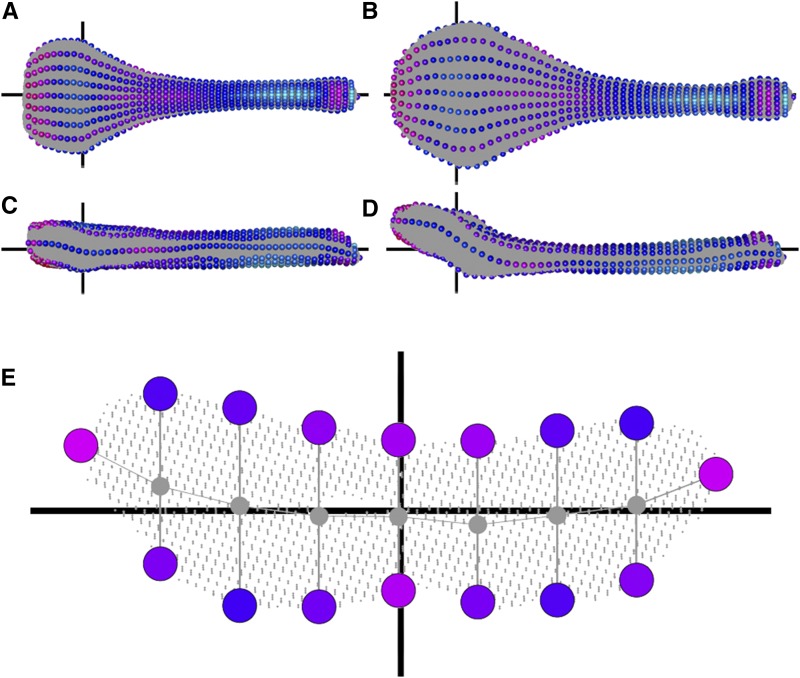
Visual representation of our morphometric pipeline for defining semilandmarks showing the two “parental extremes.” See text for details. Dorsal (A and B) and lateral (C and D) views of a typical D2 (A and C) or B6 (B, D) individual (specimens #DBA_1 and #C57_1, respectively). The gray background of each bone represents approximately 175,000 x-y-z points segmented from micro-CT scans. Moving from proximal (left sides of A–D) to distal (right sides of A–D), we sampled 50 slices. One slice is shown in more detail (E), which is looking down the center of the bone, displaying an empty internal medullary cavity. Exactly seven points were defined on the ventral and dorsal surfaces, as well as the leftmost and rightmost, totalling 16 semilandmarks per slice, indicated by spheres. The colors of the spheres indicate their contribution to shape differences (LD1) between D2 and B6 with red indicating regions that differ, and blue indicate more similar regions in comparisons between the two parental strains. Horizontal black axes indicate the z-axis (A–D) or the x-axis (E). Vertical black axes indicate the x-axis (A and B), or the y-axis (C–E).

For all specimens, the glans penis was cut proximal to the baculum, then placed in distilled water at room temperature for approximately 1 wk, at which point tissue was easily teased away using forceps and pressure of liquid flow from a squeeze bottle of 70% ethanol. Bacula were soaked in 200 μL of 1% ammonia solution for a maximum of 2 hr to clean any remaining tissue and remove oils, then dried and packed into thin layers of Aquafoam (Kokomo, IN) for micro-CT scanning. There is a distinct mass of fibrocartilage distal to the bony baculum; our study did not include this structure. For the remainder of the manuscript, “baculum” refers to the single bone in the baculum, excluding the distal fibrocartilage (Figure 1).

### Micro-CT scanning of bacula

Aquafoam slices containing bacula were stacked in a 35 mm cylindrical micro-CT sample holder. Micro-CT scans were acquired using a uCT50 scanner (Scanco Medical AG, Bruttisellen, Switzerland) at the USC imaging core facility, under the following settings: 90 kVp, 155 uA, 0.5 mm Al filter, 750 projections per 180° (360° coverage), exposure time of 500 msec, and voxel size of 15.5 μm.

### 3D transformation to “align” bacula

Customized scripts were written in Python (www.python.org) and R (R Core Team 2014) to process microCT images, segment individual bacula, perform transformations, and call semilandmarks; all scripts and microCT images have been deposited in the Figshare Data repository (DOI:10.6084/m9.figshare.3080725.v1). Each baculum was segmented out of the z-stack of micro-CT images and converted to a 3D point cloud of x-y-z points. Each pixel in the point cloud represents bone, and the images include internal structures, not just the surface. Point clouds were transformed in three main steps borrowing methodology from Dines *et al.* (2014) and are graphically illustrated in Figure 1. First, the two points furthest apart in the point cloud were used to initially define a distal-proximal axis, which we consider a z-axis. Second, the 10% most proximal and the 10% most distal points were sliced out separately, and the centroids of their convex hulls calculated. The point cloud was then transformed such that the proximal centroid became 0, 0, 0 and the distal point cloud became 0, 0, z (where z was some positive value). This second transformation effectively controlled for slight variation associated with defining points in the first step. Third, we sliced out points that fell 15.00–15.25% along the length of the new z-axis and computed its minimum bounding rectangle (MBR) (Butterworth 2013). The entire point cloud was then retransformed so that the long end of this MBR ran parallel to a new x-axis, and the short end parallel to a new y-axis. Slight curvatures in the bone, especially toward the distal end, were exploited to ensure the dorsal-ventral axis was correct. All bone transformations were visually confirmed using the RGL library in R with customized R scripts.

### Defining semilandmarks

The baculum lacks any true landmarks, so we mathematically defined 802 points along each bone (DOI:10.6084/m9.figshare.3080725.v1). These points can be thought of as “semilandmarks” (Mitteroecker and Gunz 2009; Bookstein 1997, 1991) since they correspond to regionally homologous regions across all specimens. Fifty point slices, each with thickness 0.25% the length of the z-axis, were evenly spaced along the anterior-posterior axis. One such slice is shown in detail in Figure 1E. Each slice was divided along the x-axis by seven lines running parallel to the y-axis. Points within 4% of the width of the slice to each line were projected onto that line, then the ventralmost and dorsalmost points defined as semilandmarks, specifically labeled according to slice and position. The leftmost and rightmost points of each slice were also defined as semilandmarks, for a total of 16 semilandmarks per slice (Figure 1E), and a total of 800 semilandmarks across the 50 slices. The single anterior-most and single posterior-most points of the baculum were added, bringing the total to 802 (Figure 1, A–D).

### Quantifying size variation

Size was quantified as centroid size, the square root of the sum of squared distances of these 802 semilandmarks from their centroid. Counting the number of pixels per scan, or even the density of pixels (number of pixels divided by centroid size) yielded identical results, but we present those based on centroid size for simplicity.

### Quantifying shape variation

We quantified shape difference between all possible pairs of bacula in a Generalized Procrustes framework, which standardized each set of 802 semilandmarks to a common size, translated them to a common origin, then optimally rotated their coordinates to minimize their Procrustes distance (Rohlf and Bookstein 1990; Slice 2007). During Procrustes superimposition, semilandmarks were allowed to “slide” along the bones’ surfaces using the function gpagen in the R package geomorph (Adams and Otárola-Castillo 2012), which improves alignment of corresponding anatomical regions lacking individual landmark homology (Bookstein 1997, 1996; Mitteroecker and Gunz 2009; Gunz *et al.* 2005). In short, sliding semilandmarks accompany uncertainty in specific placement of landmarks.

The gpagen analysis resulted in a pairwise distance matrix (in the case of the BXD RILs, a 369 × 369 matrix; in the case of the LGxSM AILs, a 144 × 144 matrix). Our goal was to define a single metric that summarized each specimen’s shape in the context of the parental strains. Therefore, using only specimens of the parental strains (in the case of BXD RILs, 27 B6, and 18 D2 individuals; in the case of LGxSM AILs, 10 LG, and 9 SM individuals), we performed a linear discriminant analysis on the pairwise distance matrix, using the lda function in the R package mass (Ripley 2002) and parental strain as the separating factor. With two parental strains, a single linear discriminant function exists. We then projected the entire pairwise distance matrix into the space defined by this LD1, using the predict function.

### Repeatability

To assess the repeatability of our micro-CT scanning and computational geometric manipulations, we scanned 34 bacula 81 times (32 bacula scanned twice, one baculum scanned six times, and one baculum scanned 11 times), each time removing the specimen and reloading it into Aquafoam and the micro-CT holder. For size, we calculated repeatability as the 1 – the median coefficient of variation of centroid size (unbiased standard deviation divided by the mean) across specimens. For shape, we could not calculate repeatability of LD1 because the measure spans 0, meaning we could be dividing by 0 to calculate coefficients of variation. Instead, we took the pairwise shape distance of each replicate bone to the other 368 bones in the dataset, then calculated the coefficients of variation per pairwise comparison, averaging across pairwise comparisons.

### Environmental input

To assess the effect of environment, we focused on 28 B6 individuals (10 from the Jackson Laboratory, nine from the Levitt Lab at USC, nine from the Williams/Lu Lab) and 17 D2 (seven from the Jackson Laboratory, 10 from the Levitt Lab at USC). We performed an ANOVA with lab origin nested within strain for these 45 parental individuals. There were no other strains for which we sampled substantial number of individuals from different labs.

### Heritability

Heritability, the proportion of phenotypic variance explained by genetic variance of size and shape measurements was estimated using a one-way ANOVA to summarize the amount of variance explained by strain. Among our 73 BXD RILs, we collected at least three males for 60. Heritabilities were estimated by focusing on these 60 (though the QTL mapping described below included all 73 RILs). The proportion of variance explained by strain identity was taken as the heritability.

### Mapping QTL

We employed two main analyses to map loci affecting baculum size (centroid size) and shape (LD1). For the BXD RILs, phenotypic values were first averaged for any RILs for which we had sampled multiple individuals. Genotypes were downloaded from GeneNetwork, and included 3805 markers (2.2% mean missing data per RIL) spaced at a mean 0.54 cM throughout the genome. We kept the 2953 markers that were also part of a relatively recent effort to update the mouse genetic map (Cox *et al.* 2009). After removing parental strains (which are uninformative since they lack recombinant chromosomes), we employed the scanone function in the R package qtl, using Hayley-Knott regression to estimate the location and the effect of QTL (Broman and Sen 2009). To determine significance, we permuted phenotypes and genotypes 1000 times, and took the 95th quantile of the 1000 maximum LOD scores as our empirical significance threshold. We estimated the confidence intervals of any significant QTL using the lodint function in the R package qtl, by dropping 1.5 LOD units from the maximal LOD unit on the chromosome. Age and weight could not be included as covariates in analyses of the BXD RILs because not all labs that donated carcasses collected this information, and where they did occur they often varied dramatically given the individuals derived from very diverse research programs. Ignoring age and weight should only add noise to our analysis and inflate Type II errors.

Unlike the BXD RILs, the LGxSM AILs were not maintained via brother-sister mating, so no two individuals were genetically identical but were also not equally related (Darvasi 1998; Darvasi and Soller 1995). The LGxSM AILs were analyzed using the R package qtlrel (Cheng *et al.* 2011), which accounts for background genetic relatedness prior to scanning for QTL. Genetic relatedness was estimated using all markers except the markers on the same chromosome as the one being analyzed (the “marker” option). Unlike the BXD RILs, age and weight could be included as covariates in our analyses of LGxSM AILs, because all individuals derived from the Cheverud Lab and were reared under identical conditions. Individuals were genotyped at 4716 diagnostic markers (0.1% mean missing data per specimen) spaced at a mean 0.36 F2 cM (J. M. Cheverud, unpublished data). Significance thresholds were estimated with 1000 permutations. Recombination distances were calculated from Cox *et al.* (2009).

### RNA sequencing

To potentially narrow down QTL identified in the BXD RILs to candidate genes, we generated RNA-seq data from 10 5-wk-old B6 (N = 5) and D2 (N = 5) individuals. For logistical reasons, our B6 bacula were derived from C57BL/6N, which is a substrain that is over 99.9% genetically identical to C57BL/6J. The baculum is still not fully ossified at this point (Glucksmann *et al.* 1976), so genes expressed may ultimately affect its size and shape. Focusing on 5-wk-old males admittedly overlooks potentially important expression patterns that occur prior to or after this age. Therefore, we view these data as a means to “generate” hypotheses rather than definitively link genes under QTL to causality. Because we did not identify any significant QTL among the LGxSM AILs (see below), we only gathered RNA-seq data from the BXD parental strains.

Bacula were homogenized in liquid nitrogen, then placed immediately into Trizol according to the Direct-zol RNA MiniPrep (Zymo Research R2052) protocol. RNA integrity was verified by Experion analysis (Bio-Rad). PolyA selection was carried out using Illumina Truseq V2 polyA beads. Libraries were prepared using Kapa Biosystems Stranded mRNA-Seq kit. We performed 12 PCR cycles to amplify the libraries, which were then visualized by Bioanalyzer analysis (Agilent) and quantified by qPCR (Kapa Biosystems Illumina Library Quantification Kit). Sequencing was performed on a NextSeq 500 using V2 chemistry.

Paired-end 100 bp sequencing reads were mapped using Tophat (Trapnell *et al.* 2009) and aligned to the complete *Mus musculus* GRCm38.73 genome release available from Ensembl. Tophat v2.06 was run on a Linux x86 64-bit cluster with the following –read-edit-dist 8 –read-mismatches 8 –segment-mismatches 3 –min-anchor-length 12 –report-secondary-alignments. In addition, the GRCm38.73 General Transcript File (GTF file) was included with the “-G” and “–no-novel juncs” tags, ensuring that only known annotated exons were used. These mappings of full length and junction reads were subsequently used by Cufflinks (Trapnell *et al.* 2009) to generate gene counts. Cufflinks v2.1.0 was run on a Linux x86 64-bit cluster with the following parameters “–multi-read-correct–upper-quartile-norm–compatible-hits-norm–frag-bias-correct” and with the gene annotation included in the GTF file.

Illumina sequencing generated 276 M paired end reads, of which 247 M (90%) mapped reads were used to access expression for the entire annotated transcriptome. A total of 13,926 genes had an FPKM value of at least 1 in at least one of the 10 specimens analyzed. Of these, 479 (610) were significantly differentially expressed at a corrected *P* = 0.05 (0.10) between the D2 and B6 parents.

Because our main interest with the RNA-seq data was to generate hypotheses, we were willing to accept increased Type I errors (false rejection of a null hypothesis) in favor of reduced Type II errors (false acceptance of null), so we used a cutoff of *P* = 0.10 instead of the more traditional *P* = 0.05. From the RNA-seq data, we identified differentially expressed genes (*P* = 0.10 between parental strains after correction by Benjamini-Hochberg) as well as genes that were highly expressed in both parents (in the top 10% of genes expressed with FPKM at least 1). Even though this latter category may not show evidence of differential expression between parental strains, they could still lead to differential baculum development between parental strains, for example through posttranslational modification.

### Bioinformatics

To further identify candidate genes under QTL, we characterized genetic variants that differed between the parental strains D2 and B6. Single nucleotide polymorphisms were downloaded using the Biomart tool from Ensembl version 81 (www.ensembl.org) and we identified genes with at least one nonsynonymous difference between B6 and D2 that also fell under our QTL. We also downloaded their estimates of dN/dS from “one-to-one” orthologs between mouse and rat to scan for genes with unusual rates of nonsynonymous evolution.

### Data availability

Data and associated code from the entire pipeline described below are available on the Figshare Digital Repository (DOI:10.6084/m9.figshare.3080725.v1).

## Results

We microCT-scanned 369 bacula representing 73 different BXD RILs and the two parental strains B6 and D2. Repeatability was 0.99 for centroid size, and 0.97 for shape divergence, respectively. Lab origin did not significantly influence shape (F_3,1_ = 1.3, *P* = 0.28), but did contribute to size (F_3,1_ = 12.68, *P* < 0.0001), explaining 18% of the variance in the latter case. In summary, our methods show good repeatability and any contribution arising from lab origin should not introduce systematic biases into our analyses.

### BXD RILs, size

**Figure 2:**
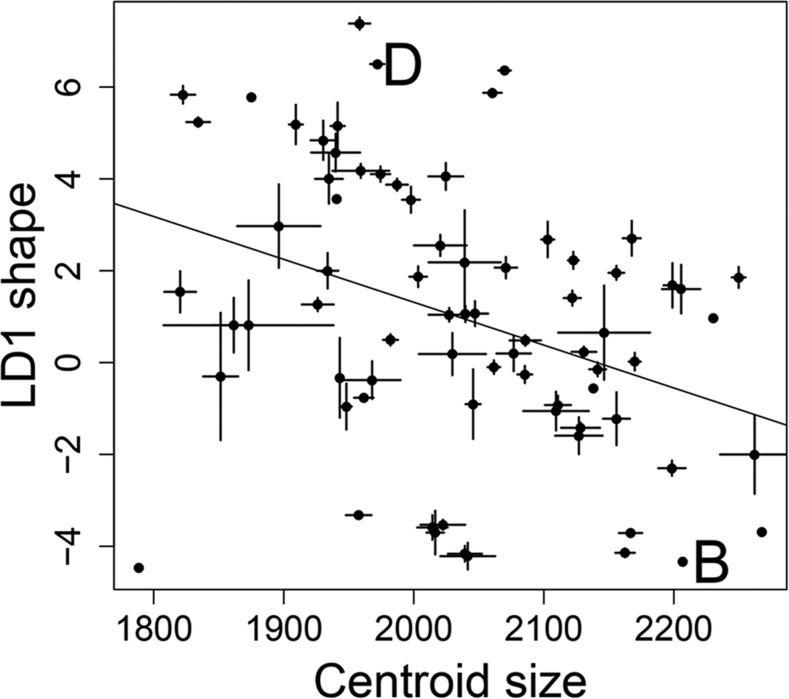
Baculum size and shape variation among the 73 BXD recombinant inbred lines (RILs) and the two parental strains D2 and B6. Each point represents the mean size and shape for each strain, bars indicate standard errors where possible. B and D indicate the two parental strains of the BXD RILs. B = B6, D = D2.

Strain was a significant predictor of size variation (F_59,244_ = 14.8, *P* < 0.01), accounting for 78% of the variance (heritability = 0.78), with BXD RILs largely falling between the parental B6 and D2 strains indicative of mostly additive genetic variance (Figure 2). Phenotype means that fell outside either parent suggested transgressive segregation or epistasis.

**Figure 3:**
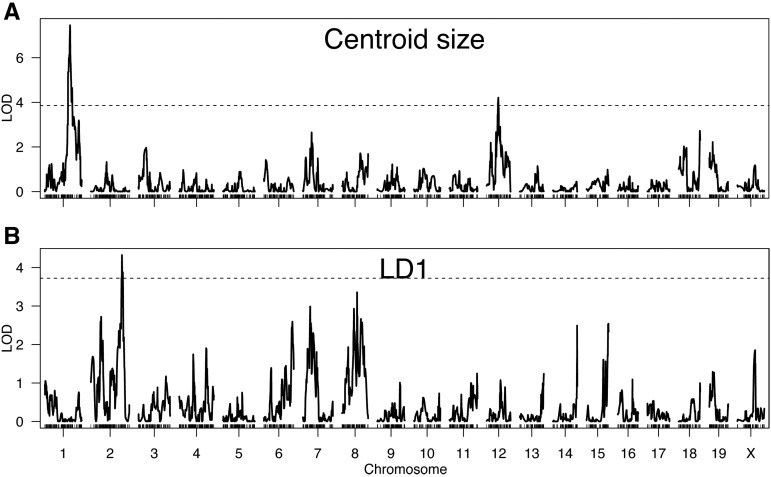
Results of scanone analyses for (A) baculum size (centroid size) and (B) shape (LD1) in the BXD recombinant inbred lines RILs. Dashed line indicates significance threshold determined from 1000 permutations of genotype and phenotype, lines indicate LOD (logarithm of the odds) scores testing the null hypothesis of no QTL (quantitative trait loci) along 2,953 markers in the genome.

**Figure 4:**
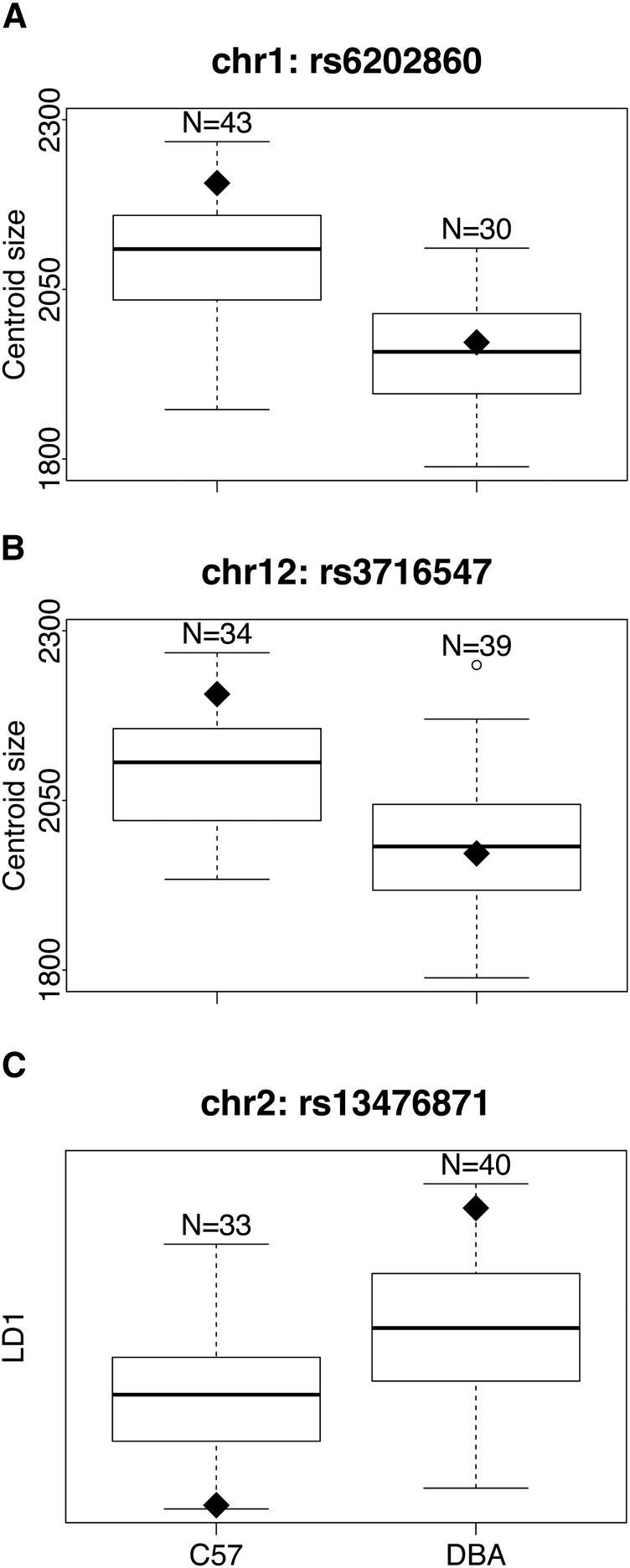
Baculum size (centroid size) and shape (LD1) from the 73 BXD recombinant inbred lines (RILs). The 73 BXD RILs are separated according to which parental allele they carry at the three QTL (quantitative trait loci) identified in Figure 3. The black diamonds represent the phenotypic value at each marker for the parental strains, C57 or DBA respectively. (A) rs6202860, the marker closest to the size QTL on chromosome 1. (B) rs3716547, the marker closest to the size QTL on chromosome 12. (C) rs13476871, the marker closest to the shape QTL on chromosome 2.

From the 73 BXD RILs, we detected two QTL for size (Figure 3A). One occurred on chromosome 1 between 136.3–150.1 Mb, with a maximum LOD score of 7.45, which was higher than all 1000 permutations (95% quantile = 3.63). The other occurred on chromosome 12, 58.0–76.4 Mb, with a maximum LOD score of 4.21, which was greater than 988 permutations (*P* = 0.012). The two markers closest to the maximal QTL peaks on chromosomes 1 or 12 showed a clear difference in centroid size among BXD RILs carrying a B6 allele *vs.* D2 allele (Figure 4, A and B). These two size QTL were not in linkage disequilbrium (χ2 = 0.16, df = 1, *P* = 0.7), as expected since they reside on different chromosomes.

### BXD RILs, shape

Strain was a significant predictor of LD1 variation (F_59,244_ = 18.1, *P* < 0.01), accounting for 81% of the variance (heritability = 0.81), with BXD RILs largely falling between the parental B6 and D2 strains indicative of mostly additive genetic variance (Figure 2). From the 73 BXD RILs, we detected a single QTL on chromosome 2, 154.7–161.7 Mb, with a maximum LOD score of 4.32, which was higher than 988/1000 permutations (*P* = 0.012) (Figure 3B). At the marker closest to the maximum QTL peak of chromosome 2, there was a clear difference in LD1 between the 33 BXD RILs with a B6 allele compared to the 40 with a D2 allele (Figure 4C). This shape QTL was not in linkage disequilibrium with either of the two size QTL (χ2 = 0.01, 0.32, df = 1, *P* = 0.9, 0.6, respectively), as expected since they reside on different chromosomes.

### BXD RILs, size and shape correlated

Interestingly, the mean size and shape per strain were correlated (Pearson’s correlation coefficient = –0.35, *P* = 0.002, Figure 2), even though major-effect QTL affecting size and shape were found on different chromosomes. This correlation suggests that small bacula tend to have the straight shape of D2, while large bacula tend to have the dorsoventral curvature of B6.

### BXD RILs, RNA-seq and bioinformatics

There were 111 (162) protein-coding genes that occurred under the size QTL identified on chromosome 1 (chromosome 12), of which 93 (145) were expressed, 3 (5) were highly expressed, 1 (2) was differentially expressed, and 11 (14) had at least one nonsynonymous variant between B6 and D2 (Table S1 and Table S2).

There were 160 protein-coding genes that occurred under the shape QTL on chromosome 2, of which 140 were expressed, 12 highly expressed, 2 differentially expressed, and 27 had at least one nonsynonymous variant (Table S1 and Table S2).

A literature review of all protein-coding genes that fell under any one of our three QTL (Figure 3) and were also highly expressed, differentially expressed, or had at least one nonsynonymous variant, revealed 16 genes with potentially interesting effects on bone morphology (Table S3). Four of these – *Ptprc*, *2700049A03Rik*, *Aspm*, and *Kif14* – showed relatively high pairwise dN/dS estimates when compared to their one-to-one ortholog in rat (all above the 75% genome-wide quantile).

### LGxSM AILs, size and shape

From the LGxSM AILs, we microCT-scanned 144 bacula, including 36 unrelated F43 and 89 F44 from 52 unique families, as well as 10 LG and 9 SM parental strains. Although both centroid size and LD1 variance showed evidence of largely additive genetic contribution (Figure S1), no significant QTL were identified for either (Figure S2). Furthermore, there was no correlation between the proportion of an individual’s genome that was from the LG parent and either baculum size (centroid size) or shape (LD1) (Pearson’s correlation coefficient, *P* = 0.98, 0.67, respectively). This is probably an indication that our sample size for LGxSM was underpowered, a hypothesis supported by the lack of significant QTL or correlation between proportion LG/J alleles and body size (Pearson’s correlation coefficient, *P* = 0.75), a trait that is highly heritable in larger studies of LGxSM intercrosses (Kramer *et al.* 1998; Cheverud *et al.* 1996).

## Discussion

Baculum size and shape diverge rapidly across species, with known effects on male reproductive success. By combining a novel morphometric pipeline with the power of mouse quantitative genetics, we make three main discoveries. First, a few QTL of major effect influence baculum variation in the BXD RILs, with two QTL explaining 50.6% of the variance in size, and a third QTL explaining 23.4% of the variance in shape. Interestingly, no QTL were detected in a second genetic system, the LGxSM AILs. Second, these major-effect QTL affecting size were distinct from the major-effect QTL affecting shape, even though the two traits were correlated. Third, by combining our QTL with RNA-seq data as well as bioinformatic features and literature review, we identified 16 promising candidate genes that may enable a deeper understanding of the genetic basis of their rapid divergence.

### Testing predictions that stem from rapid divergence

Our finding of a few QTL with large effects suggests that morphological divergence of the baculum may accumulate easily, acting via mutations in a few key targets. Several studies (all in insects, most in *Drosophila*) have found QTL of large effect (>10% of phenotypic variance) on aspects of the male genital apparatus. It should be emphasized that like the current study, none of these studies have estimated effect sizes from specific genes. Many of these studies were initiated by crossing two closely related species, then backcrossing the F1s to either parental species, including two species of carabid beetles (Sasabe *et al.* 2007), *Drosophila simulans* and *D. mauritiana* (True *et al.* 1997; Zeng *et al.* 2000; Liu *et al.* 1996; LeVasseur-Viens and Moehring 2014; Tanaka *et al.* 2015), *D. simulans* and *D. sechellia* (Macdonald and Goldstein 1999), and *D. yakuba* and *D. santomea* (Peluffo *et al.* 2015). Although large-effect QTL were detected in all these studies, the extent of linkage that will exist in these backcross designs, coupled with the limited number of markers used to interrogate the genome, may underestimate the true number of QTL and thus inflate the percent variance explained by any single QTL. Furthermore, it is possible that interspecific studies are biased toward finding large-effect QTL since the different species employed were already known to harbor highly divergent male genitalia. Alternatively, we might predict a bias toward finding small-effect QTL if genetic effects are masked by disruption of normal development in hybrid males.

In contrast, studies within species tend to find QTL with smaller effects. Three QTL contributed 4.7–10.7% to variance in the shape of the posterior lobe of genital arch within an advanced intercross panel of *D. melanogaster* (McNeil *et al.* 2011). Similarly, a genome-wide association test among 155 inbred strains of *D. melanogaster* strains of the *Drosophila* Genetic Resource Panel (Mackay *et al.* 2012) found multiple SNPs throughout the genome that correlated with small to moderate differences in the size and shape of the posterior lobe (Takahara and Takahashi 2015). Another study in *D. montana* showed mostly small effect QTL explaining less than 10% of the variance (Schäfer *et al.* 2011). Taken together, QTL identified in these intraspecific studies are more numerous, with smaller effect sizes, than the interspecific studies discussed above. In this sense, our study is unusual in that it was an intraspecific study but identified a few QTL of large effect, although it should be noted that a small amount of interspecific introgression was detected in both B6 and D2 (Yang *et al.* 2011). In contrast, a recent morphometric study of mouse skulls found many QTL of very small effect (Maga *et al.* 2015). Perhaps bacula are unique in that a few major QTL enable their rapid divergence.

In our study, QTL affecting size and shape were independent of each other, another genetic characteristic that might allow for more rapid morphological divergence, since mutations may be “less pleiotropic.” Size and shape QTL could not be separated in many of the studies mentioned above (Liu *et al.* 1996; Macdonald and Goldstein 1999), as the low number of markers employed precluded delineation of multiple QTL. Two studies within *D. melanogaster* found distinct QTL affecting size *vs.* shape of male posterior lobes (Schäfer *et al.* 2011; Takahara and Takahashi 2015). Interestingly, we found that the major QTL affecting size and shape of the baculum were independent of each other (Figure 3), even though size and shape were correlated (Figure 2). One explanation is that many QTL of small effect were not detected here but sufficient to drive an overall correlation between size and shape.

Two additional predictions could not be properly evaluated here. First, we predicted that different genetic backgrounds of mice might yield distinct QTL, meaning there are multiple genetic pathways that can generate baculum variation. Some support for this hypothesis comes from a comparison of two quantitative genetic studies that mapped nonoverlapping QTL in two different populations of *D. melanogaster* (Takahara and Takahashi 2015; McNeil *et al.* 2011). However, one study of a *D. mauritiana–D. simulans* backcross identified identical QTL as found in a *D. mauritiana–D. sechellia* backcross (LeVasseur-Viens and Moehring 2014). In the current study, we found three large-effect QTL in the BXD RILs (Figure 3), none of which were found in the LGxSM AIL panel (Figure S2). However, the lack of QTL in the LGxSM AIL panel may simply be due to lack of power.

Second, we predicted that baculum variation might arise via alterations in gene expression since both early genital development and bone morphogenesis proceed from highly conserved molecular pathways where protein-coding changes would be expected to have dire consequences (Carroll 2008). Our data do not yet speak to this question; across the 16 candidate genes identified under our three QTL (Table S3), seven were highly or differentially expressed among parental strains of the BXD RILs, and 10 had at least one nonsynonymous mutation. Further evaluation of whether baculum variation arises from expression or structural prediction requires finer scale mapping, more detailed expression studies, and/or stronger evidence that certain genes are good candidates for baculum variation.

### Rapid genital divergence *vs.* conserved genital development pathways

One of the paradoxes of the rapid evolution of male genitalia, at least in vertebrates, is the highly conserved set of genes that appear to be expressed during early genital development, including Sonic Hedgehog (*Shh*), various fibroblast growth factor receptors (*Fgfr’s*), and various homeobox-containing genes of the D cluster (*Hoxd*) (Haraguchi *et al.* 2001, 2000; Klonisch *et al.* 2004; Perriton *et al.* 2002; Miyagawa *et al.* 2009; Cohn 2011; Seifert *et al.* 2009; Gredler *et al.* 2015; Sanger *et al.* 2015; Infante *et al.* 2015). In fact, the outgrowth of external genitalia shares many genetic pathways with the development of digits (Warot *et al.* 1997; Tschopp *et al.* 2014; Lonfat *et al.* 2014; Cobb and Duboule 2005; Haraguchi *et al.* 2000; Kondo *et al.* 1997; Perriton *et al.* 2002; Dollé *et al.* 1991; Zákány and Duboule 1999) or gut (Cohn 2011), although genital-specific enhancers modify their expression (Lonfat *et al.* 2014).

*Hoxd-13* mutant mice have smaller bacula than wild-type mice (Hérault *et al.* 1997; Zákány and Duboule 1999), and differently shaped bacula (Peichel *et al.* 1997), and *Hoxd* genes have enhancers that drive expression specific to external genitalia (Dollé *et al.* 1991; Lonfat *et al.* 2014). When *Hoxd-13*, *Hoxd-11*, and *Hoxd-12* were all experimentally made nonfunctional, no genitals developed (Kondo *et al.* 1997). However, all these genetically manipulated individuals show extreme dysmorphia in body plan, and many are lethal before birth. Importantly, none of the QTL that we observed here overlap with *Shh*, *Fgfr*’s, or various *Hoxd* genes, suggesting the variation we observe is not due to genetic variation in these conserved pathways, although it is formally possible that something under our QTL affects expression of these genes in *trans*.

### Candidate genes

Our study offers the first set of candidate genes to explain variation in baculum size and shape. Although systematically testing which (if any) of these protein-coding genes explain baculum variance remains outside the scope of the current study, we prioritized genes based on three criteria: 1) they fell under QTL identified above (Figure 3), 2) they were highly expressed or differentially expressed in 5-wk-old bacula, or had at least one nonsynonymous variant between parental strains, and 3) literature searching revealed a potential link to bone or genital morphogenesis. The baculum forms after birth, through both direct ossification and ossification of cartilage anlage as the animal approaches sexual maturity (Murakami and Mizuno 1984), so bone-related phenotypes are potentially interesting. By these criteria, we identified 16 candidate genes (Table S3).

Four genes under the size QTL of chromosome 1 are potentially interesting candidates. *Kif14* and *Aspm* have been shown to cause cranial deformities and reduced body size (Fujikura *et al.* 2013; Pulvers *et al.* 2010). A third, *Ptprc*, alters bone morphology when knocked out (Shivtiel *et al.* 2008). Finally, mice deficient in *Mgat2* exhibit reduced body size and reduced bone density due to hyperactive osteoclasts (Wang *et al.* 2001).

Under the size QTL of chromosome 12, *Dact1* has been shown to negatively regulate *Wnt* signaling (Wen *et al.* 2010), which in turn affects the development of bone and genitals (Zheng *et al.* 2012; Berendsen and Olsen 2015; Hu *et al.* 2005; Haraguchi *et al.* 2000, 2001; Yamaguchi *et al.* 1999). *Wnt* signaling has also been implicated in the divergence of male genitalia in flies (Tanaka *et al.* 2015). Mice missing a functional *Dact1* allele lacked external genitals and displayed numerous skeletal abnormalities (Wen *et al.* 2010). Another gene under this QTL, *Hif1a*, also affects bone density (Wang *et al.* 2007).

Two genes under the shape QTL of chromosome 2 influence bone development. *Rbl1* is a key regulator of cell development which, if inactivated, results in shortened limbs, defective endochondral ossification, altered chondrocyte growth (Cobrinik *et al.* 1996), and reduced body size (Scimè *et al.* 2005). A second gene, *Tgm2*, plays a role in chondrogenesis and ultimately bone formation (reviewed in Iismaa *et al.* 2009).

### Conclusions

Our study provides the first candidate genes to explain variation in baculum size and shape, a structure with an astonishing rate of evolutionary divergence. Our study provides evidence that baculum variation is explained by a few QTL of large effect that independently affect size and shape with apparently minimal pleiotropic side-effects, and may help reconcile the paradox of rapid morphological divergence coupled with conserved developmental pathways. Future experiments should focus on testing candidate genes identified here as a means to genetically dissect its function, and to provide a deeper understanding of the rapid evolution of this amazing structure.

## Supplementary Material

Supplemental Material
