# Supplementary material for: The Genetic Basis of Baculum Size and Shape Variation in Mice
Source: G3 (Bethesda). 2016 Mar 1;6(5):1141–51. doi: 10.1534/g3.116.027888 (PMC4856068; doi:10.1534/g3.116.027888)
Supplement: Supplemental Material [file supp_6_5_1141__index.html]

The Genetic Basis of Baculum Size and Shape Variation in Mice — Supplemental Material 

# The Genetic Basis of Baculum Size and Shape Variation in Mice

## Supplemental Material for Schultz *et al.*

**Files in this Data Supplement:**

- Figure S1 - Phenotypic distribution of baculum size (centroid size, upper panel) and shape (LD1, lower panel) from LGxSM AILs. Green indicates parental strains (in upper panel SM on left, LG on right; in lower panel, LG on left, SM on right), blue indicates F43 individuals and red indicates F44 individuals. Error bars are the standard error (unbiased standard deviation divided by the mean) for any families where multiple individuals were phenotyped. (PDF, 13 KB)
- File S1 - https://figshare.com/articles/SuppFile1\_tar\_gz/3080725

  This tar.gz contains the scripts and .xyz point clouds from Schultz et al.
- Figure S2 - Scans for QTL in the LGxSM for size (centroid size) and shape (LD1) failed to yield any significant correlations. Chromosomes labeled along x-axis, LOD scores on y-axis. (JPG, 1 MB)
- Table S1 - Expression values (FPKM) for all genes detected in 5-week-old bacula. Column headers with DBA in the name are the DBA individuals. Column headers with TGM4 in the name are C57 individuals. FC=fold change. 626 p.value=significance associated with the Benjamini-Hochberg-corrected exact test in EdgeR. (.xlsx, 3.3 MB)
- Table S2 - Protein coding genes that fall under one of the three QTL identified in the BXD family, and also had at least one nonsynonymous variant between C57 and DBA. (.csv, 5 KB)
- Table S3 - Genes that fell under one of the three QTL identified in the BXD family and were highly expressed or differentially expressed between C57 and DBA. (.xlsx, 55 KB)
