# Supplementary figures and images for: The Genetic Basis of Baculum Size and Shape Variation in Mice
Source: G3 (Bethesda). 2016 Mar 1;6(5):1141–51. doi: 10.1534/g3.116.027888 (PMC4856068; doi:10.1534/g3.116.027888)

## Size

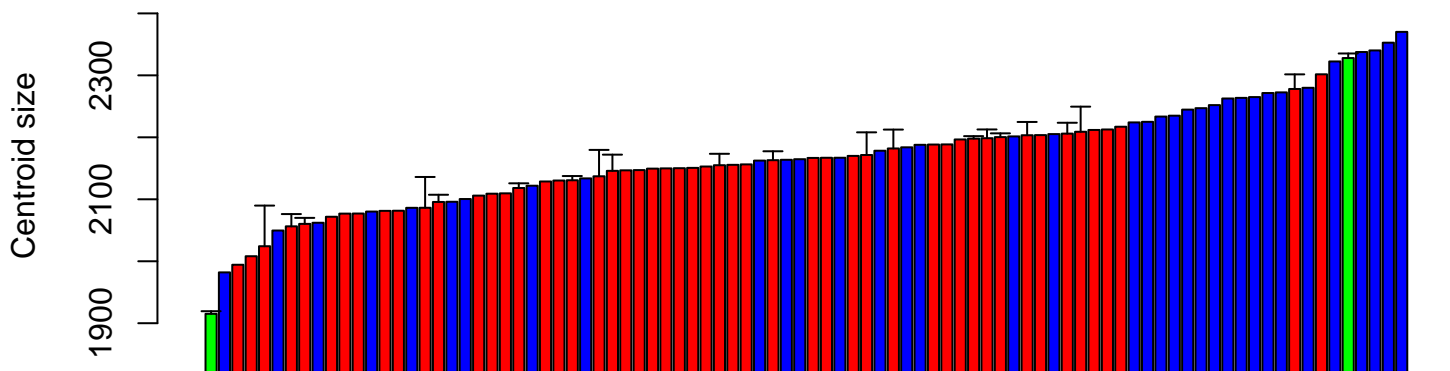

## Shape

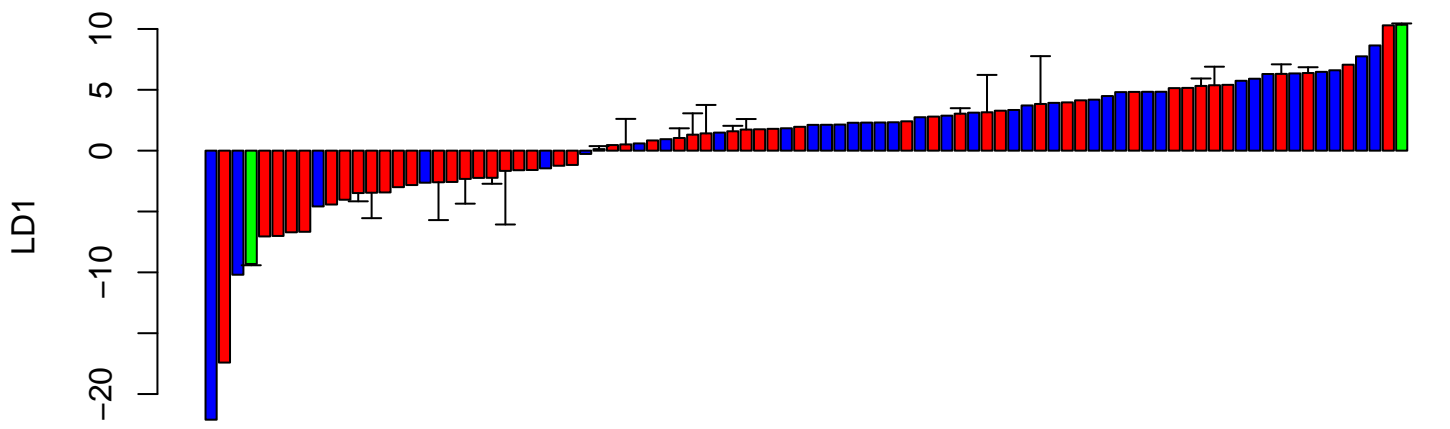

Supplement: Supplemental Material [file supp_g3.116.027888_SuppFig1.pdf]

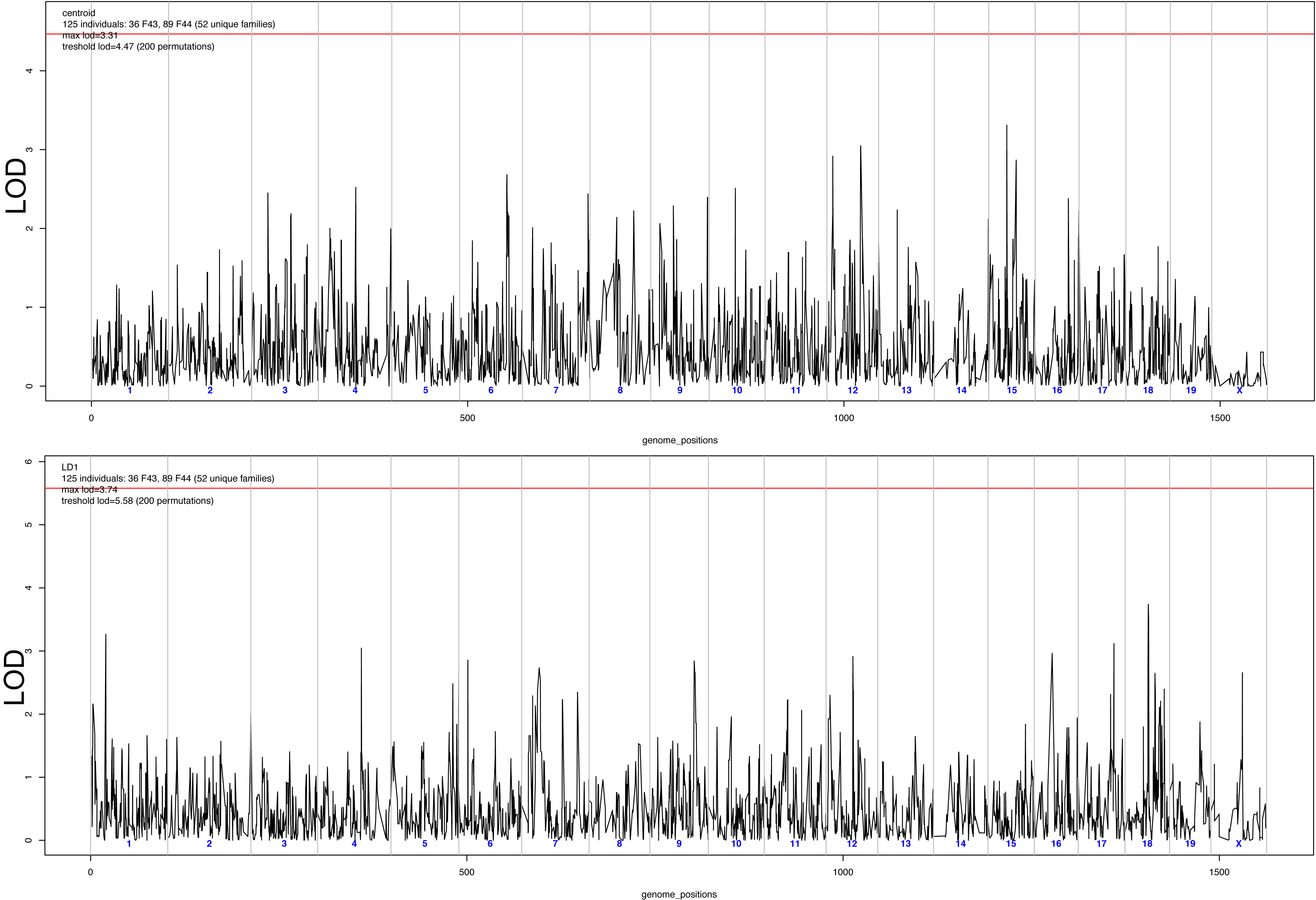

Supplement: Supplemental Material [file supp_g3.116.027888_SuppFig2.jpg]
