# Supplementary material for: The Genetic Basis of Baculum Size and Shape Variation in Mice
Source: G3 (Bethesda). 2016 Mar 1;6(5):1141–51. doi: 10.1534/g3.116.027888 (PMC4856068; doi:10.1534/g3.116.027888)
Supplement: Supplemental Material [file supp_g3.116.027888_FileS1.pdf]

G3 Genes | Genomes | Genetics
Genetics Society of America
9650 Rockville Pike
Bethesda, MD 20814-3998
www.genetics.org
www.genestogenomes.org
genetics-gsa@thegsajournals.org

https://figshare.com/articles/SuppFile1_tar_gz/3080725

This tar.gz contains the scripts and .xyz point clouds from Schultz et al
